# Supplementary material for: Paroxysmal sympathetic hyperactivity during neurorehabilitation for severe acquired brain injury: current Scandinavian practice and Delphi consensus recommendations
Source: BMJ Open. 2024 May 28;14(5):e084778. doi: 10.1136/bmjopen-2024-084778 (PMC11138287; doi:10.1136/bmjopen-2024-084778)
Supplement: Author's manuscript [file bmjopen-2024-084778.draft_revisions.pdf]

BMJ Open

BMJ Open is committed to open peer review. As part of this commitment we make the peer review history of every article we publish publicly available.

When an article is published we post the peer reviewers' comments and the authors' responses online. We also post the versions of the paper that were used during peer review. These are the versions that the peer review comments apply to.

The versions of the paper that follow are the versions that were submitted during the peer review process. They are not the versions of record or the final published versions. They should not be cited or distributed as the published version of this manuscript.

BMJ Open is an open access journal and the full, final, typeset and author-corrected version of record of the manuscript is available on our site with no access controls, subscription charges or pay-per-view fees (http://bmjopen.bmj.com).

If you have any questions on BMJ Open's open peer review process please email info.bmjopen@bmj.com

BMJ Open

Paroxysmal Sympathetic Hyperactivity during Neurorehabilitation for severe acquired brain injury: Current Nordic Practice and Delphi Consensus Recommendations

Journal:	*BMJ Open*
Manuscript ID	bmjopen-2024-084778
Article Type:	Original research
Date Submitted by the Author:	28-Jan-2024
Complete List of Authors:	Godbolt, Alison; Karolinska Institute Department of Clinical Sciences Danderyd Hospital, Dept of Clinical Sciences and University Dept of Rehabilitation Medicine<br>Zampakas, Alexandros; Danderyd University Hospital, Department of Rehabilitation Medicine<br>Nygren Deboussard, Catharina; Karolinska Institute Department of Clinical Sciences Danderyd Hospital, Dept of Clinical Sciences and University Dept of Rehabilitation Medicine
Keywords:	Brain Injuries, REHABILITATION MEDICINE, Neurological injury < NEUROLOGY

SCHOLARONE™
Manuscripts

I, the Submitting Author has the right to grant and does grant on behalf of all authors of the Work (as defined in the below author licence), an exclusive licence and/or a non-exclusive licence for contributions from authors who are: i) UK Crown employees; ii) where BMJ has agreed a CC-BY licence shall apply, and/or iii) in accordance with the terms applicable for US Federal Government officers or employees acting as part of their official duties; on a worldwide, perpetual, irrevocable, royalty-free basis to BMJ Publishing Group Ltd ("BMJ") its licensees and where the relevant Journal is co-owned by BMJ to the co-owners of the Journal, to publish the Work in this journal and any other BMJ products and to exploit all rights, as set out in our licence.

The Submitting Author accepts and understands that any supply made under these terms is made by BMJ to the Submitting Author unless you are acting as an employee on behalf of your employer or a postgraduate student of an affiliated institution which is paying any applicable article publishing charge ("APC") for Open Access articles. Where the Submitting Author wishes to make the Work available on an Open Access basis (and intends to pay the relevant APC), the terms of reuse of such Open Access shall be governed by a Creative Commons licence – details of these licences and which Creative Commons licence will apply to this Work are set out in our licence referred to above.

Other than as permitted in any relevant BMJ Author's Self Archiving Policies, I confirm this Work has not been accepted for publication elsewhere, is not being considered for publication elsewhere and does not duplicate material already published. I confirm all authors consent to publication of this Work and authorise the granting of this licence.

For peer review only

Paroxysmal Sympathetic Hyperactivity during Neurorehabilitation for severe acquired brain injury: Current Nordic Practice and Delphi Consensus Recommendations

Alison K. Godbolt*[1,2], Alexandros Zampakas[1], Catharina Deboussard[1,2]

*corresponding author. Email: alison.godbolt@ki.se

1. University department of Rehabilitation Medicine, Danderyd hospital, Stockholm, Sweden.

2. Department of Clinical Sciences, Karolinska Institute Danderyd Hospital, Stockholm, Sweden

Word count:

Abstract 196 words

Body of text 4476 words

For peer review only

Abstract:

Objectives: To document current practice and develop consensus recommendations for the assessment and treatment of PSH during rehabilitation after severe acquired brain injury

Design: Delphi consensus process with three rounds, based on the CREDES guidelines, led by three convenors (the authors) with an expert panel. Round one was exploratory, with consensus defined before round two as agreement of at least 75% of the panel.

Setting: A working group within the Nordic Network for Neurorehabilitation.

Panel Participants: Twenty specialist physicians, from Sweden (9 participants), Norway (7) and Denmark (4), all working clinically with patients with severe acquired brain injury and with current involvement in clinical decisions regarding PSH.

Results: Consensus was reached for 21 statements on terminology, assessment and principles for pharmacological and non-pharmacological treatment, including some guidance on specific drugs. From these, an algorithm to support clinical decisions at all stages of inpatient rehabilitation was created.

Conclusions: Considerable consensus exists in the Nordic countries regarding principles for PSH assessment and treatment. An interdisciplinary approach is needed. Improved documentation and collation of data on treatment given during routine clinical practice are needed as a basis for improving care until sufficiently robust research exists to guide treatment choices.

Strengths and limitations of this study:

Strengths:

- Robust process for forming consensus according to published recommendations
- Involvement of clinicians working at all stages of inpatient rehabilitation
- Panel representation from three Nordic countries and most health care regions within countries

Limitations:

- insufficient number of panel participants with experience of care after discharge from inpatient rehabilitation for consideration of this phase
- panel participants required to have clinical experience of PSH during the past year, but no specific requirement on number of patients treated by panel participants

Keywords: Traumatic brain injury, acquired brain injury, paroxysmal sympathetic hyperactivity, rehabilitation, assessment, management, treatment

For peer review only - http://bmjopen.bmj.com/site/about/guidelines.xhtml

INTRODUCTION:

Following severe acquired brain injury some patients develop episodes of excessive sympathetic activity that can be very pronounced, with tachycardia, tachypnoea, fever, motor posturing, sweating and hypertension. The 2014 publication(1) of consensus on definitions, nomenclature and diagnostic criteria was a major step forward regarding this syndrome that had previously been referred to by a very large number of different terms. Paroxysmal Sympathetic Hyperactivity (PSH) as defined in 2014 has become the recommended and established term for this syndrome.

Subsequent studies(2) have shown an association of PSH with longer length of hospital stay and with more unfavourable outcomes. Experts in neurocritical care have published articles(2, 3) suggesting treatment options, but a recent scoping review(4) found a lack of evidence to inform clinical decision making for patients with paroxysmal sympathetic hyperactivity (PSH) after severe acquired brain injury.

Many studies and reviews of PSH have originated from authors working within neurointensive care(2, 3). Whilst acknowledging the value of these publications, there is also a need for rehabilitation considerations and the rehabilitation care process to be considered more explicitly to support optimal care continuing into care settings following neurointensive care, for those patients with continuing PSH later after injury. Sedating drugs that may be appropriate for use for patients who are anaesthetized on the neurointensive care unit may become inappropriate as rehabilitation priorities gradually evolve.

In clinical practice, patients may also be transferred between some (or even many) different wards and rehabilitation units during their inpatient care. It is a challenge to create management strategies that are sufficiently flexible to be appropriate on different units with very different staffing and availability of monitoring, and at the same time to be sufficiently clear to support good care when the total number of patients with PSH that individual clinicians in some care settings treat is small. A recent study that is from a neurorehabilitation setting (5), on patients with Prolonged Disorders of Consciousness (the most severely injured subgroup of all with PSH), noted a prevalence of PSH of 31% in this subgroup at rehabilitation admission, suggesting that PSH is a not inconsiderable issue.

Consensus recommendations have a place in supporting best clinical practice for today's patients, until adequate research evidence can be produced. A Delphi process is an established, structured technique for formation of consensus based on four methodological aspects(6): a group of experts ("the panel") answer questions, the process is anonymous to ensure that less dominant views come forward, the process is iterative, with several rounds of questions, and subsequent rounds are based on a summary of group responses to the previous round.

The aim of the project was to develop consensus recommendations for assessment and treatment of PSH during rehabilitation after severe acquired brain injury.

METHODS:

The CREDES recommendations(6) for Delphi processes were used as the basis for the consensus process.

Justification for use of a Delphi process: Uncertainties regarding best treatment for PSH and the lack of adequate research findings to assist physicians in clinical decisions were discussed at a meeting of the Brain Injury Pharmacotherapy Special Interest Group of the Nordic Network for Neurorehabilitation(7) at a meeting in Oslo in November 2022. A decision was taken to hold a Delphi process to establish the degree of consensus.

Planning and process: During the spring of 2023, the convenors (the authors) created a project plan and questions for the first round based on previous discussions of areas of difficulty and published,

For peer review only - http://bmjopen.bmj.com/site/about/guidelines.xhtml

peer reviewed articles on PSH. These were discussed with an independent Rehabilitation physician and Neurosurgeon (see acknowledgements) from a different region to the convenors, following which adjustments were made to the project plan and questions. Before round one, Consultant physicians in Rehabilitation medicine at the authors' department, who were not participants in the panel, answered a pilot version and gave feedback on the question wording and content before final adjustments. The Delphi process was run between August and December 2023; Round one was distributed in August, round two in October and round three in December. A summary of responses was sent to the panel after completion of each round and before distribution of the next round.

Establishing the panel: Factors of importance regarding choice of panel participants were discussed by the convenors before sending invitations. Representation from each health care region in Denmark, Sweden and Norway was sought, with an aim of 1 to 3 panel participants from each region. We sought panel participants who were physicians who had completed specialist training, worked clinically with patients with severe acquired brain injury in the Nordic countries, and had been involved in clinical decisions about drugs treatment of PSH in the previous year; these formed criteria for participation. Potential panel participants were identified via lists of participants in the Nordic Network of Neurorehabilitation meeting in November 2022, by network contacts, and by contact with the authors of a recent Nordic publication on PSH(8). Invited participants received an email with information about the project, extent of panel involvement, and confirmed that they met the above criteria for panel participants prior to answering round one.

Patient group: Patients with severe acquired brain injury defined as any sudden insult to a previously essentially healthy brain in a patient aged at least 2 years, of a severity that requires initial neurointensive care. Diagnoses include traumatic brain injury, anoxic brain injury, subarachnoid haemorrhage, spontaneous intracerebral haemorrhage, cerebral infarction.

Rehabilitation focus: The focus of this study was on patients undergoing rehabilitation after severe acquired brain injury, including early rehabilitation which may occur during neurointensive care, intermediary care, care on an acute ward or inpatient care on a rehabilitation unit. Due to the focus on rehabilitation this survey did not include patients who are anaesthetized and mechanically ventilated in the very early stages after brain injury, during which phase other treatments may be relevant.

Definition of consensus: Round one was an exploratory round with the aim of finding an optimal focus for the rest of the process. The decision was therefore made (and communicated to panel participants) before round one that criteria for consensus would be decided after completion of round one. Panel participants received the following information on stopping criteria before they answered round two: "Statements that 75% of the panel agree with will be considered to represent consensus in the Nordic countries (Denmark, Sweden, Norway) at this time. The conveners will consider the development of responses and the likelihood of coming to consensus in specific aspects after round 3, and then consider whether further rounds (maximum 5) will give added value. If after 3 rounds we have not reached consensus for some items, and it is unlikely that consensus will be reached, areas where at least 50% of the panel agree will also be reported as areas of considerable agreement (but not consensus)."

Procedure for the rounds:

<u>Round one</u> included questions on panel participants' speciality and clinical experience, and on a wide range of aspects of PSH assessment and management. Questions were in various forms, where closed questions (possible answers, "yes, no, I don't know") were complemented with free text comment boxes. Round one also included questions about drugs used for treatment of PSH. A list was given based on 29 classes of drugs (with examples) reported as being used for PSH a recent scoping review (4). For each phase of care that they participated in, panel participants were asked to indicate all drugs they used, also the three drugs most often used, and comment on reasons for their

For peer review only - http://bmjopen.bmj.com/site/about/guidelines.xhtml

use or non-use. Phases of care were included due to expected differences in treatment priorities with care setting and were defined as follows: 1. Breathing spontaneously with no or only light sedation on the neurointensive care unit. The patient may have respiratory support. 2. Breathing spontaneously with no or only light sedation on an intermediate care unit / high dependency unit. The patient may have respiratory support. 3. Cared for on an acute care ward or rehabilitation ward at an acute hospital. The patient may have respiratory support. 4. Cared for on a rehabilitation ward without acute hospital facilities. 5. Cared for outside hospital (home, nursing home).

Round two: Based on collated responses to round one, a number of statements were created for round two, which the panel were asked to respond to with "agree, disagree" or, "I don't know". For each statement they could also comment and suggest changes. The five drugs that were included in at least 25% of the panels' lists of the three most commonly used drug treatments for PSH at any phase of care, were carried forward to round two. Panel participants were asked, for each phase of case which they participated in, to indicate how commonly they prescribed these drugs for PSH on a scale of 0 (never) to 50 (for about half of patients) to 100 (always).

Round three included reformulated statements for the minority of areas where consensus had not been reached in round two, with a new possibility to agree or disagree. Round three also included possible refinements for three statements where consensus had already been reached in round two, but where individual panel participants had suggested refinements or improvements in their responses to round two. Panel participants were in round three asked to confirm their agreement or otherwise with the original version of these statements from round two, state their agreement or otherwise to the adjusted versions of these statements, and finally to express a preference for one version.

Platform: REDCap(9) is a secure web application for building and managing online surveys and databases. REDCap was used to produce and send out the questionnaires for each round, to send pre-scheduled and extra reminders for submission of answers, and also to distribute summaries of findings from each round before the following round. Personalised email reminders were also sent to panel participants who had not responded by the initial deadline, with extensions given of up to a week.

Consent: Panel participants gave consent for participation in the panel, storage of email addresses and de-identified responses, feedback of anonymised responses to the panel during the process, and publication of study findings.

RESULTS:

Panel participants: Of 23 physicians invited to participate, 20 met the criteria, formed the panel, and responded to rounds one and two. 19 of these also responded to round three. Four panel participants worked with patients in Denmark, seven panel participants in Norway and nine in Sweden. Medical specialities of panel participants were Rehabilitation Medicine or equivalent for 14, Intensive care medicine for two, Neurosurgery for three and neurology for one. Three of the Rehabilitation medicine specialists were also specialists in another area: two in neurology, and one in intensive care medicine. Panel participants had a mean of 10.3 years of clinical experience of acquired brain injury (standard deviation 6,7). Panel participants were from 2 of 4 Danish Health Care regions, 4 of 5 Norwegian health care regions and 5 of 6 Swedish Health care regions.

Panel participants' clinical involvement at the different phases of care: 6 panel participants were involved with decisions regarding PSH treatment for patients breathing spontaneously with no or light sedation on the neurointensive care unit, 10 on the intermediate care (or high dependency) unit, 12 during care on an acute or rehabilitation ward at an acute hospital, 9 during care on a rehabilitation ward without acute hospital facilities, and 3 for patients cared for outside hospital (at

For peer review only - http://bmjopen.bmj.com/site/about/guidelines.xhtml

home or in an nursing home). Some panel participants were involved during several phases of care. The number of panel participants with current experience of patients care for outside hospital was considered too few for formation of consensus and this phase of care was not included in subsequent rounds.

Process: Consensus was reached for many areas in round 2. A third round was run to allow consideration of refinements of some statements and to consider alternatives to two statements where consensus had not been reached in round 2. The project was concluded at the end of round three.

Consensus statements: At least 75% of panel participants agreed to the following statements by the end of round three. Two statements concerned only the later phases of the chain of care. For these statements, only responses from panel participants who worked during the later phases of care were considered when calculating percentage agreement. Following each statement, the number of panel participants who agreed, disagreed or didn't know is given, and the round after which consensus was reached for the final wording is given.

1. The term paroxysmal sympathetic hyperactivity (PSH) is the preferred term for use in clinical practice. The diagnosis should be made according to the consensus definition of Baguley et al 2014. As described by Baguley et al, PSH may occur after severe acquired brain injury, including but not limited to traumatic brain injury. *Agree 19, Disagree 0, Don't know 1. Consensus after round 2.*

2. Differential diagnoses should be considered and as far as possible excluded and/or treated before making the diagnosis of PSH. *Agree 20, Disagree 0, Don't know 0. Consensus after round 2.*

3. The PSH assessment measure (PSH-AM) should be used to support the initial clinical diagnosis and management of PSH. In some clinical settings aspects of the PSH-AM may be difficult to implement and additional or alternative supporting tools may need to be developed. *Agree 17, Disagree 1, Don't know 2. Consensus after round 2.*

4. PSH will in most cases have developed and been diagnosed during neurointensive care or intermediary care. In those cases where the diagnosis has not been made, the PSH assessment measure is also recommended for use on acute wards or inpatient rehabilitation units. *Responses only sought from panel participants working on acute wards or inpatient rehabilitation units, n=14: Agree 14, Disagree 0, Don't know 0. Consensus after round 3.*

5. For patients who have a diagnosis of PSH made on the neurointensive or intermediary care ward and where drug treatment for PSH is ongoing when they are discharged, there is a need for follow up later in the chain of care. The PSH-assessment measure may be used during inpatient rehabilitation as part of follow up and to guide decisions on tapering of treatment. *Responses only sought from panel participants working on acute wards or inpatient rehabilitation units, n=14: Agree 14, Disagree 0, Don't know 0. Consensus after round 3.*

6.The effectiveness of prophylactic drug treatment for patients with a risk of PSH is unknown. Negative effects of drug treatment are difficult to evaluate in patients with severe acquired brain injury. At the current time prophylactic pharmacological treatment aimed at preventing PSH in patients who do not have a PSH diagnosis cannot therefore be recommended. *Agree 19, Disagree 0, Don't know 1. Consensus after round 2.*

7. General principles for treatment: Choice of PSH drug therapy should take into consideration phase of recovery, care setting, and current medical and rehabilitation priorities such as need to promote recovery of consciousness and cognition. *Agree 20, Disagree 0, Don't know 0. Consensus after round 2.*

For peer review only - http://bmjopen.bmj.com/site/about/guidelines.xhtml

8. When initiating treatment of PSH, treatment should aim to treat both sympathetic hyperactivity and to minimize the most prominent symptoms of PSH in the individual patient. *Agree 19, disagree 0, don't know 0. Consensus after round 3.*

9.PSH-triggers should be assessed, and measures taken to minimize these before and during any pharmacological treatment. PSH-triggers may include urinary retention, constipation, gastric paresis, spasticity, poor positioning (where spasticity may contribute), allodynic responses to poorly fitting orthotics, pressure sores and other causes of pain, environmental factors such as ambient temperature and noise. Units should develop routines for a structured approach to minimization of triggers. *Agree 20, Disagree 0, Don't know 0. Consensus after round 2.*

10. Supportive treatment (hydration, reduction of fever, correction of electrolytes, nutrition) should be optimized in parallel to pharmacological treatment for PSH. Delays in pharmacological treatment should be avoided in moderate to severe cases. Units should develop routines for a structured approach to optimization of supportive treatment. *Agree 17, Disagree 1, Don't know 1. Consensus after round 3.*

11. PSH as the treatment indication should be clearly documented in the medical record when prescribing drugs to treat PSH, when transferring patients between units, and at discharge. This is particularly important as many drugs used for PSH also have other indications. Treatment response to any current or previous PSH drug treatment should also be documented. Future medication review later in the chain of care should be planned. *Agree 19, Disagree 0. Don't know 1. Consensus after round 2.*

12. Sedating drugs should be minimized wherever possible when treating PSH, due to negative effects on recovery of consciousness and cognition, risk for other negative effects of sedation such as retained respiratory secretions, and impact on participation in rehabilitation. This is especially relevant in rehabilitation settings but should also be considered during neurointensive care and intermediary (high dependency) care. For very severe cases short periods of treatment with sedating drugs may be unavoidable. *After round 3: Agree 18, Disagree 1, Don't know 0. Consensus on a previous version in round 2, with final consensus on this alternative wording in round 3.*

13. The efficacy and relatively favourable side effect profiles of non-selective beta blockers and Gabapentin make these appropriate for first line treatment of PSH for most patients after neurointensive care. *After round 3: Agree 17, Disagree 0, Don't know 1, no answer 1. Consensus reached in round 2 and confirmed after rejection of an alternative wording in round 3.*

14. Opiates are often necessary for PSH early after injury due to good effects on many PSH components. However, they should be tapered when possible later during rehabilitation due to negative effects on alertness and cognition. *Agree 20, Disagree 0, Don't know 0. Consensus after round 2.*

15. Use of benzodiazepines primarily for treatment of PSH should be minimized (regarding dose and treatment duration) during phases in recovery when the focus is rehabilitation, due to negative effects on cognition and alertness. Their use may be necessary in the initial treatment of PSH on the neurointensive care unit and/or high dependency unit but they should be tapered when possible. *Agree 20, Disagree 0, Don't know 0. Consensus after round 2.*

16. Botulinum toxin may be needed for treatment of focal spasticity generally after severe acquired brain injury, and specifically for patients where focal or regional spasticity contributes to triggering of PSH-episodes. *Agree 19, Disagree 0, Don't know 1. Consensus after round 2.*

17. Enteral baclofen may be necessary for PSH with considerable disturbances of muscle tone, but negative effects on cognition and alertness may contribute to difficulties with participation in

For peer review only - http://bmjopen.bmj.com/site/about/guidelines.xhtml

rehabilitation. These negative effects should be considered when prescribing and during follow up. *Agree 20, Disagree 0, Don't know 0. Consensus after round 2.*

18. An intrathecal baclofen pump should be considered for severe PSH where other measures have not been effective. *Agree 16, Disagree 1, Don't know 3. Consensus after round 2.*

19. PSH abates (reduces or disappears) with increased time after injury for most patients. Drug and other treatments for PSH should therefore be reviewed regularly and reduced or ceased if and when possible. *Agree 20, Disagree 0, Don't know 0. Consensus after round 2.*

20. The course of PSH in the post-acute phases (on the rehabilitation ward and beyond) and optimal tapering of medication when episodes abate are insufficiently documented and should be a focus of future research and development work. *Agree 15, Disagree 0, Don't know 2, No answer 3. Consensus after round 2.*

21. Ethical challenges in treating PSH in patients who often lack the capacity to consent to treatment are acknowledged. When the patient is unable to participate in treatment discussions next of kin and/or legal representatives should be informed of treatment decisions and involved in discussions of the often difficult balance between negative effects of PSH and potential negative effects of treatment (side effects, sedation). The presence of PSH is also associated with more severe brain injury with poorer outcomes, about which relatives should be informed. *Agree 19, Disagree 1, Don't know 0. Consensus after round 2.*

Choice of Drugs for PSH-treatment

In round one the panel indicated which drugs they use with a primary purpose of treating PSH. To support recall of any less usual choices, and to aid analysis, panel participants were presented with a list of 29 drug classes with examples from a 2021 Scoping Review on PSH(4) and asked to indicate all drugs they used: there was an unlimited number of choices, and no restrictions were made at this stage regarding phase of recovery. Drugs used were (drug, number of panel participants): Opiates (15), non-selective β-blockers (14), Gabapentionoid e.g. Gabapentin (14), GABA-B agonist e.g. Baclofen (11), α2 agonist e.g. Clonidine (10), Paracetamol (10), selective β-blockers (4), antiepileptics (3), antihistamine (3), Benzodiazepines (3), Other anesthetics (3), Adamantane e.g. Amantadine (2) Sedative hypnotics (2), Barbiturates (1), Dopamine agonist (1), Hyrdrazinophalazine (1), Ibuprofen (1), Muscle relaxant (1), Others (0).

In round two, panel participants indicated how often they used the drugs that had been most commonly cited in round one, for each of the phases of care in which they were involved (see table), by moving a horizontal slider for each drug. The five drug classes for which responses were sought, and given in the table, were those which in round one were included in at least 25% of individual panel participants' lists of their three most used drugs, at any stage of care.

------ insert table near here -------

For peer review only - http://bmjopen.bmj.com/site/about/guidelines.xhtml

Algorithm: The consensus statements and panel particpants' drug choices are summarised in the algorithm in the figure, which is intended to support clinicians in their assessment and management of patients with PSH. The algorithm was presented to the panel in round three with resulting comments incorporated into the final version --------- *insert figure near here* ---------.

DISCUSSION:

After an initial exploratory round in this Delphi process on the management of PSH during rehabilitation after severe acquired brain injury, consensus was reached for 18 statements in round two, on terminology, assessment, non-pharmacological management, and treatment principles. Consensus for three further statements and refined wording for two statements were achieved in round three. Responses on drug choices were less clear cut: panel participants' drug choices at the various phases of care show clear trends, but also a considerable variability in practice. This was not unexpected given the state of the evidence (weak). Alpha-2 agonists and opiates are used most in the early stages of recovery, on the neurointensive care and intermediary care units, and infrequently later during inpatient care and rehabilitation, although some panel participants continue to use these drugs even at these later stages. Non-selective beta-blockers are most commonly used at all phases of care after neurointensive care, and Gabapentin is also commonly used in these phases. Panel feedback also highlighted certain management difficulties due to non-patient factors. For example although consensus was reached that an intrathecal baclofen pump should be considered for the most severe, refractory cases, this may be unavailable in some centres.

A number of the consensus statements are of a confirmatory nature, reiterating key aspects of the literature on PSH and putting these into a clinical context for routine practice. For example, although it will soon be 10 years since the publication of consensus on terminology and diagnosis, initial discussions and feedback to round one showed that other terms remain in use in today's clinical practice in the Nordic countries. Some statements also make explicit aspects that might be considered obvious components of good care, but where panel particpants' clinical experiences are of common difficulties (for example non-tapering of drugs as PSH abates). It is hoped that this broad Nordic consensus on recommended terminology, assessment and treatment principles will support good clinical practice for patients with PSH. Other consensus statements begin to address common difficulties encountered in later clinical management of patients who may need to be transferred between units several times before leaving inpatient care. The panel's experience is that indications for each prescribed drug are not always specified at the time of transfer between units, which creates difficulties in follow up and ongoing optimisation of drug treatment.

Strengths of the consensus process include the following of published recommendations for Delphi processes, an explorative first round, clear differentiation in questions to panel participants of the various phases of care (from neurointensive care to a rehabilitation ward outside an acute hospital), inclusion of panel participants from several medical specialities, inclusion of non-pharmacological aspects of assessment and management, and broad geographical representation from Denmark, Norway and Sweden. Limitations include an insufficient number of panel participants with experience of care after discharge from inpatient rehabilitation for consideration of this phase, and lack of data on the extent of panel participant's clinical experience of PSH; although it was a requirement that panel participants had been involved in clinical decisions for patients with PSH during the past year, there was no specific requirement regarding the number of patients treated by panel participants.

We consider that this Delphi process adds to previous studies by giving more explicit consideration to changing priorities through the rehabilitation chain of care, and clarifies the necessity of an interdisciplinary approach to assessment and management involving the rehabilitation team, at all phases including very early rehabilitation. An algorithm has been included (see figure) as a summary

For peer review only - http://bmjopen.bmj.com/site/about/guidelines.xhtml

of the consensus statements, with the intention of assisting physicians in treatment of patients with PSH in all phases of inpatient care after early sedation on the neurointensive care unit has been reduced.

Whilst robust research studies of the effects of drugs on PSH are desirable, the clinical complexity of most patients affected by PSH and its relative rarity are expected to make definitive studies difficult to perform. Studies evaluating real-world data from treatment of patients carried out during routine clinical practice are also needed to improve treatment. For this to be possible, better structures are needed for assessment, diagnosis, treatment and follow up. “Structures” include routines guided by these consensus criteria, also consideration of medical journal systems and coding; there is no ICD-10 diagnosis for paroxysmal sympathetic hyperactivity which is a hinder to data assimilation from clinical practice. There are also examples of limitations in electronic medical journal systems preventing documentation of paroxysmal sympathetic hyperactivity as a drug indication as the term has more characters than allowed in some prescribing systems. Such organisational and system factors should receive more attention in future studies and planning for care of patients with severe acquired brain injury. Continued educational efforts about PSH are also needed to inform the less specialised health care staff that the patient is likely to encounter with increased time after brain injury.

CONCLUSION:

A considerable degree of consensus has been reached in the Nordic countries for the clinical assessment and management of patients with PSH after severe acquired brain injury. Uncertainties remain about specific drug choices, which need to be modified in pace with changing rehabilitation priorities.

REFERENCES:

1. Baguley IJ PI, Fernandez-Ortega JF, Rabinstein AA, Dolce G, Hendricks HT. Paroxysmal sympathetic hyperactivity after acquired brain injury: consesnsus on conceptual definition, nomenclature, and diagnostic criteria. Journal of Neurotrauma. 2014;1;31(17):1515-20.
2. Geert Meyfroidt IJB, David K Menon. Paroxysmal sympathetic hyperactivity: the storm after acute brain injury. Lancet Neurology. 2017;16(9):721-9.
3. Rui-Zhe Zheng Z-QL, Run-Ze Yang, Guo-Hui Huang, Guang-Ming Zhang. Identification and Management of Paroxysmal Sympathetic Hyperactivity After Traumatic Brain Injury. Frontiers of Neurology. 2020;11:81.
4. Justin Siew Yoong Tu JR, Adam M Deane, Mark P Plummer. Pharmacological Management of Paroxysmal Sympathetic Hyperactivity: A Scoping Review. Journal of Neurotrauma. 2021;38(16):2221-37.
5. Lucia F Lucca ADT, Francesca Cava, Annamaria Romoli, Rita Formisano, Federico Scarponi, Anna Estraneo, Diana Frattini, Paolo Tonin, Chiara Bertolino, Pamela Salucci, Bahia Hakiki, Mariagrazia D'Ippolito, Mauro Zampolini, Orsola Masotta, Silvia Premoselli, Matteo Interlenghi, Christian Salvatore, Annalisa Polidori, Antonio Cerasa. Predicting Outcome of Acquired Brain Injury by the Evolution of Paroxysmal Sympathetic Hyperactivity Signs. Journal of Neurotrauma. 2021;38:1988-94.
6. Saskia Jünger SAP, Jenny Brine, Lukas Radbruch and Sarah G Brearley. Guidance on Conducting and REporting DElphi Studies (CREDES) in palliative care: Recommendations based on a methodological systematic review. Palliative Medicine. 2017.
7. Neurorehabilitation NNf. [19th January 2024]. Available from: https://www.nordisk-netvaerk-neurorehab.com/formaal/.
8. Alba Corell JL. ABC om Paroxysmal sympatisk hyperaktivitet (Article in Swedish). Läkartidningen. 2021;118:21070.
9. RedCap 2024 [updated 19th January 2024. Available from: https://www.project-redcap.org/.

For peer review only - http://bmjopen.bmj.com/site/about/guidelines.xhtml

Author contributions: All authors contributed to study design, conduction of the study, and analysis of data. Drs Zampakas and Deboussard formed and distributed the surveys in REDCap. Dr Godbolt drafted the article and created a sketch for the figure. Drs Zampakas and Deboussard critically appraised and approved the article text.

Acknowledgments - We thank the following:

Doctors Per Ertzgaard, Rehabilitation Physician and Fredrik Ginstman, Neurosurgeon, both of the University Hospital Linköping, Sweden, for valuable comments on the project plan and a pilot version of the questions for round one.

The panel participants for their valuable contributions to the Delphi Consensus process: Daniel Dahlgren and Alison Godbolt, Department of Rehabilitation Medicine, Danderyd Hospital, Sweden. Sofie Jacobson, Department of Rehabilitation Medicine, Umeå University Hospital, Sweden. My Dung Nguyen Torkildsen, Head of department, Physical medicine and rehabilitation, Stavanger University Hospital, Norway. Tanja Bertheussen, Dept of Physical medicine and Rehabilitation, Kongsgård, Sørlandet Hospital, Norway. Alba Corell, Department of Clinical Neuroscience, Institute of Neuroscience and Physiology, Sahlgrenska Academy, University of Gothenburg, Gothenburg, Sweden, Department of Neurosurgery, Sahlgrenska University Hospital, Gothenburg, Sweden. Kristin Alvsaaker, Postoperative and Intensive Care Department, Oslo University Hospital (OUH), Norway and Department of Physical Medicine and Rehabilitation, OUH, Norway. Tiina Ader, Department of Physical Medicine and Rehabilitation, Haukeland University Hospital, Norway. Merete Petersen, Department of Neuroanesthesiology, Rigshospitalet, University Hospital, Denmark. Cathrine Elisabeth Einarsen, Clinic of Rehabilitation, St. Olavs Hospital, Trondheim University Hospital, Trondheim, Norway. Tove Grønbæk Jensen, Hammel neurocentre, Denmark. Marina Parziali, Neurorehabilitation, NU Health Care, Sweden. Ursula Heldmann, Rehabilitation medicine, Skane University Hospital, Sweden. Astrid Wille-Jørgensen, Dept. of Brain and Spinal Cord Injury, Bodil Eskesen Center, Rigshospitalet, Demark. Maritza Beekman, Department of Rehabilitation Medicine, University Hospital Linköping, Sweden. Marco Brizzi, Neurology, Skane University Hospital, Sweden. Caroline Ustvedt, Department of Traumatic Brain Injury, Sunnaas Rehabilitation Hospital, Norway. Shirin Kordasti Frisvold, Chief consultant, Department of Intensive Care and Department of Anesthesiology and Intensive Care, University Hospital of North Norway, Tromso. Rasmus Langelund Jørgensen, Hammel Neurorehabilitation and Research Centre, Denmark. Laura Serrano Barrenechea, Dept of Rehabilitation Medicine South Älvsborgs Hospitla and Ryhov Hospital.

Stefan Arousell, Dept of Rehabilitation Medicine, Danderyd hospital, Stockholm for graphic design support in creation of the figure.

Funding statement: This research received no specific grant from any funding agency in the public, commercial or not-for-profit sectors.

Patient and public involvement: Patients and the public were not involved.

Competing Interest Statement: Dr Godbolt is the specialty expert for Rehabilitation Medicine for the Stockholm Region. Dr Zampakas has received compensation from Ipsen for support with a workshop at the Nordic Toxins conference, October 2023. Dr Deboussard is the Chair for the National Program Group, and for the Regional Program Group for Stockholm-Gotland, for Rehabilitation Habilitation and Insurance Medicine, within the National system for knowledge-driven management within Swedish healthcare.

For peer review only - http://bmjopen.bmj.com/site/about/guidelines.xhtml

Table: Panel participants' frequency of use of common drugs for PSH

	Neurointensive care	Intermediary care	Acute Hospital – acute or rehabilitation ward	Inpatient rehabilitation outside acute hospital
respondents	*n=6*	*n=9*	*n=9*	*n=8*
**Drug:**				
Alpha-2 agonists e.g. Clonidine	80,5 [30 – 100]	50 [16-100]	23,5 [0-96]	10 [0-90]
Baclofen (enteral)	32,5 [0-89]	26,5 [0-75]	22 [0-70]	28 [12-50]
Beta blocker, non-selective, e.g. Propranolol	65 [20-94]	83 [16-100]	81 [50-100]	75,5 [50-100]
Gabapentin	38 [0-63]	50 [0–93]	60,5 [21-90]	50 [27-94]
Opiates	75 [64-85]	68 [31–99]	26 [0-70]	28 [0-96]

Legend for table: The table gives the median and [range] for how often panel members use the stated drugs to treat PSH, on a continuous scale where 0 = never, 50= half of patients, 100 = always. Note: Data characteristic suggested technical difficulties for 2 panel participants: in one case "50" was registered for all drugs in all care settings, which seems unlikely to be the intended response. In another case 0 was registered for all drugs for all care settings after intermediary care; the instruction was to not answer for care settings that respondents do not work in rather than to answer 0. These responses have been excluded from the summary above.

Figure – see separate file

Legend for figure: *PSH – paroxysmal sympathetic hyperactivity, CRS-R – coma recovery scale-revised*

For peer review only

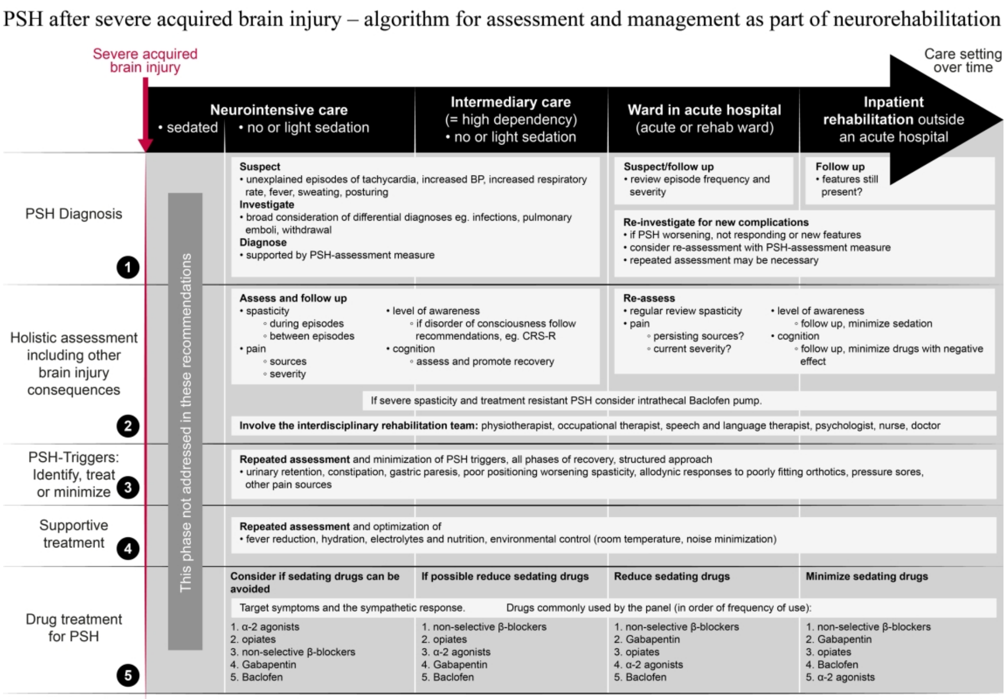

PSH – paroxysmal sympathetic hyperactivity, CRS-R – coma recovery scale-revised

268x188mm (300 x 300 DPI)

Checklist for submission to BMJ Open

Box 3. Recommendations for the Conducting and REporting of DElphi Studies (CREDES).

Rationale for the choice of the Delphi technique

1. *Justification.* The choice of the Delphi technique as a method of systematically collating expert consultation and building consensus needs to be well justified. When selecting the method to answer a particular research question, it is important to keep in mind its constructivist nature *Included*

Planning and design

2. *Planning and process.* The Delphi technique is a flexible method and can be adjusted to the respective research aims and purposes. Any modifications should be justified by a rationale and be applied systematically and rigorously *Noted*
3. *Definition of consensus.* Unless not reasonable due to the explorative nature of the study, an a priori criterion for consensus should be defined. This includes a clear and transparent guide for action on (a) how to proceed with certain items or topics in the next survey round, (b) the required threshold to terminate the Delphi process and (c) procedures to be followed when consensus is (not) reached after one or more iterations *Done*

Study conduct

4. *Informational input.* All material provided to the expert panel at the outset of the project and throughout the Delphi process should be carefully reviewed and piloted in advance in order to examine the effect on experts' judgements and to prevent bias *Done*
5. *Prevention of bias.* Researchers need to take measures to avoid directly or indirectly influencing the experts' judgements. If one or more members of the research team have a conflict of interest, entrusting an independent researcher with the main coordination of the Delphi study is advisable *No conflict of interest of relevance*

*Consolidated criteria for reporting qualitative research
**Consolidated Standards of Reporting Trials

Box 3. (Continued)

6. *Interpretation and processing of results.* Consensus does not necessarily imply the 'correct' answer or judgement; (non)consensus and stable disagreement provide informative insights and highlight differences in perspectives concerning the topic in question *Noted*
7. *External validation.* It is recommended to have the final draft of the resulting guidance on best practice in palliative care reviewed and approved by an external board or authority before publication and dissemination *No such body available.*

Reporting

8. *Purpose and rationale.* The purpose of the study should be clearly defined and demonstrate the appropriateness of the use of the Delphi technique as a method to achieve the research aim. A rationale for the choice of the Delphi technique as the most suitable method needs to be provided *Done*
9. *Expert panel.* Criteria for the selection of experts and transparent information on recruitment of the expert panel, socio-demographic details including information on expertise regarding the topic in question, (non)response and response rates over the ongoing iterations should be reported *Done*
10. *Description of the methods.* The methods employed need to be comprehensible; this includes information on preparatory steps (How was available evidence on the topic in question synthesised?), piloting of material and survey instruments, design of the survey instrument(s), the number and design of survey rounds, methods of data analysis, processing and synthesis of experts' responses to inform the subsequent survey round and methodological decisions taken by the research team throughout the process *Done*
11. *Procedure.* Flow chart to illustrate the stages of the Delphi process, including a preparatory phase, the actual 'Delphi rounds', interim steps of data processing and analysis, and concluding steps *Covered by article text*
12. *Definition and attainment of consensus.* It needs to be comprehensible to the reader how consensus was achieved throughout the process, including strategies to deal with non-consensus *Done*
13. *Results.* Reporting of results for each round separately is highly advisable in order to make the evolving of consensus over the rounds transparent. This includes figures showing the average group response, changes between rounds, as well as any modifications of the survey instrument such as deletion, addition or modification of survey items based on previous rounds *Done*
14. *Discussion of limitations.* Reporting should include a critical reflection of potential limitations and their impact of the resulting guidance *Done*
15. *Adequacy of conclusions.* The conclusions should adequately reflect the outcomes of the Delphi study with a view to the scope and applicability of the resulting practice guidance *Done*
16. *Publication and dissemination.* The resulting guidance on good practice in palliative care should be clearly identifiable from the publication, including recommendations for transfer into practice and implementation. If the publication does not allow for a detailed presentation of either the resulting practice guidance or the methodological features of the applied Delphi technique, or both, reference to a more detailed presentation elsewhere should be made (e.g. availability of the full guideline from the authors or online; publication of a separate paper reporting on methodological details and particularities of the process (e.g. persistent disagreement and controversy on certain issues)). A dissemination plan should include endorsement of the guidance by professional associations and health care authorities to facilitate implementation *Dissemination planned*

ref: Guidance on Conducting and REporting DElphi Studies (CREDES) in palliative care. Jünger et al, Palliative Medicine 2017

BMJ Open

Paroxysmal Sympathetic Hyperactivity during Neurorehabilitation for severe acquired brain injury: Current Scandinavian Practice and Delphi Consensus Recommendations

Journal:	*BMJ Open*
Manuscript ID	bmjopen-2024-084778.R1
Article Type:	Original research
Date Submitted by the Author:	19-Apr-2024

Complete List of Authors:	Godbolt, Alison; Karolinska Institute Department of Clinical Sciences Danderyd Hospital, Dept of Clinical Sciences and University Dept of Rehabilitation Medicine<br>Zampakas, Alexandros; Danderyd University Hospital, Department of Rehabilitation Medicine<br>Nygren Deboussard, Catharina; Karolinska Institute Department of Clinical Sciences Danderyd Hospital, Dept of Clinical Sciences and University Dept of Rehabilitation Medicine

**Primary Subject Heading**:	Rehabilitation medicine
Secondary Subject Heading:	Neurology
Keywords:	Brain Injuries, REHABILITATION MEDICINE, Neurological injury < NEUROLOGY

SCHOLARONE™
Manuscripts

I, the Submitting Author has the right to grant and does grant on behalf of all authors of the Work (as defined in the below author licence), an exclusive licence and/or a non-exclusive licence for contributions from authors who are: i) UK Crown employees; ii) where BMJ has agreed a CC-BY licence shall apply, and/or iii) in accordance with the terms applicable for US Federal Government officers or employees acting as part of their official duties; on a worldwide, perpetual, irrevocable, royalty-free basis to BMJ Publishing Group Ltd ("BMJ") its licensees and where the relevant Journal is co-owned by BMJ to the co-owners of the Journal, to publish the Work in this journal and any other BMJ products and to exploit all rights, as set out in our licence.

The Submitting Author accepts and understands that any supply made under these terms is made by BMJ to the Submitting Author unless you are acting as an employee on behalf of your employer or a postgraduate student of an affiliated institution which is paying any applicable article publishing charge ("APC") for Open Access articles. Where the Submitting Author wishes to make the Work available on an Open Access basis (and intends to pay the relevant APC), the terms of reuse of such Open Access shall be governed by a Creative Commons licence – details of these licences and which Creative Commons licence will apply to this Work are set out in our licence referred to above.

Other than as permitted in any relevant BMJ Author's Self Archiving Policies, I confirm this Work has not been accepted for publication elsewhere, is not being considered for publication elsewhere and does not duplicate material already published. I confirm all authors consent to publication of this Work and authorise the granting of this licence.

For peer review only

Paroxysmal Sympathetic Hyperactivity during Neurorehabilitation for severe acquired brain injury: Current Scandinavian Practice and Delphi Consensus Recommendations

Alison K. Godbolt*[1,2], Alexandros Zampakas[1], Catharina Deboussard[1,2]

*corresponding author. Email: alison.godbolt@ki.se

1. University department of Rehabilitation Medicine, Danderyd hospital, Stockholm, Sweden.

2. Department of Clinical Sciences, Karolinska Institute Danderyd Hospital, Stockholm, Sweden

Word count:

Abstract 206 words

Body of text 5552 words

For peer review only - http://bmjopen.bmj.com/site/about/guidelines.xhtml

Abstract:

Objectives: To document current practice and develop consensus recommendations for the assessment and treatment of Paroxysmal Sympathetic Hyperactivity (PSH) during rehabilitation after severe acquired brain injury

Design: Delphi consensus process with three rounds, based on the Guidance on Conducting and REporting DElphi Studies (CREDES) guidelines, led by three convenors (the authors) with an expert panel. Round one was exploratory, with consensus defined before round two as agreement of at least 75% of the panel.

Setting: A working group within the Nordic Network for Neurorehabilitation.

Panel Participants: Twenty specialist physicians, from Sweden (9 participants), Norway (7) and Denmark (4), all working clinically with patients with severe acquired brain injury and with current involvement in clinical decisions regarding PSH.

Results: Consensus was reached for 21 statements on terminology, assessment and principles for pharmacological and non-pharmacological treatment, including some guidance on specific drugs. From these, an algorithm to support clinical decisions at all stages of inpatient rehabilitation was created.

Conclusions: Considerable consensus exists in the Nordic countries regarding principles for PSH assessment and treatment. An interdisciplinary approach is needed. Improved documentation and collation of data on treatment given during routine clinical practice are needed as a basis for improving care until sufficiently robust research exists to guide treatment choices.

Strengths and limitations of this study:

- Robust process for forming consensus according to published recommendations
- Involvement of clinicians working at all stages of inpatient rehabilitation
- Panel representation from three Scandinavian countries and most health care regions within countries

- insufficient number of panel participants with experience of care after discharge from inpatient rehabilitation for consideration of this phase
- panel participants required to have clinical experience of PSH during the past year, but no specific requirement on number of patients treated by panel participants

Keywords: Traumatic brain injury, acquired brain injury, paroxysmal sympathetic hyperactivity, rehabilitation, assessment, management, treatment

For peer review only - http://bmjopen.bmj.com/site/about/guidelines.xhtml

INTRODUCTION:

Following severe acquired brain injury some patients develop episodes of excessive sympathetic activity that can be very pronounced, with tachycardia, tachypnoea, fever, motor posturing, sweating and hypertension. The 2014 publication(1) of consensus on definitions, nomenclature and diagnostic criteria was a major step forward regarding this syndrome that had previously been referred to by a very large number of different terms. Paroxysmal Sympathetic Hyperactivity (PSH) as defined in 2014 has become the recommended and established term for this syndrome. Similar symptoms may also occur due to a broad range of other complications of brain injury or co-morbidities, including but not limited to infections, heterotopic ossification, alcohol or drug withdrawal. Alternative or contributing diagnoses must be identified and (when necessary) treated. A diagnosis of PSH is made with consideration of the clinical features above, together with diagnostic likelihood after consideration of differential diagnoses (1).

Subsequent studies(2) have shown an association of PSH with longer length of hospital stay and with more unfavourable outcomes. Experts in neurocritical care have published articles(2, 3) suggesting treatment options, but a recent scoping review(4) found a lack of evidence to inform clinical decision making when treating patients with PSH after severe acquired brain injury.

Mechanisms underlying PSH remain incompletely understood and are likely to be complex. One prominent model is the excitatory/inhibitory ratio model (2, 5), whereby impairment of central descending inhibition of sympathetic and motor pathways leads to loss of control of the excitatory action of spinal circuits. This explains pathological sympathetic responses to minimally or non-nociceptive stimuli, such as tracheal suction or placement of orthotics. A central autonomic network subserves this central descending inhibition, comprising both cortical and subcortical components (6). Whilst certain structures (midbrain, pons, periventricular white matter, corpus callosum (2)) seem to have a key role in PSH, it is the overall severity of brain injury (rather than specific anatomical areas of injury) that likely gives an increased risk of PSH. Increased catecholamine (epinephrine, norepinephrine, dopamine) and ACTH levels have been demonstrated in traumatic brain injury patients during PSH episodes (7), adding support to theories of sympathetic over-activity. Other mechanisms have also been proposed, although with only limited underlying research: rat studies have suggested putative roles of neutrophil extracellular traps (traumatic brain injury model (8)), and hydrogen sulfide (cerebral infarct model (9)). Published treatment suggestions (2, 4) are based to varying extents on targeting of such putative mechanisms.

Many studies and reviews of PSH have originated from authors working within neurointensive care (2, 3). Whilst acknowledging the value of these publications, there is also a need for rehabilitation considerations and the rehabilitation care process to be considered more explicitly to support optimal care continuing into care settings following neurointensive care, for those patients with continuing PSH later after injury. Sedating drugs that may be appropriate for use for patients who are anaesthetized on the neurointensive care unit may become inappropriate as rehabilitation priorities gradually evolve.

In clinical practice, patients may also be transferred between some (or even many) different wards and rehabilitation units during their inpatient care. It is a challenge to create management strategies that are sufficiently flexible to be appropriate on different units with very different staffing and availability of monitoring, and at the same time to be sufficiently clear to support good care when the total number of patients with PSH that individual clinicians in some care settings treat is small. A recent study that is from a neurorehabilitation setting (10), on patients with Prolonged Disorders of Consciousness (the most severely injured subgroup of all with PSH), noted a prevalence of PSH of 31% in this subgroup at rehabilitation admission, suggesting that PSH is a not inconsiderable issue.

For peer review only - http://bmjopen.bmj.com/site/about/guidelines.xhtml

Consensus recommendations have a place in supporting best clinical practice for today's patients, until adequate research evidence can be produced. A Delphi process is an established, structured technique for formation of consensus based on four methodological aspects(11): a group of experts ("the panel") answer questions, the process is anonymous to ensure that less dominant views come forward, the process is iterative, with several rounds of questions, and subsequent rounds are based on a summary of group responses to the previous round.

The aim of the project was to develop Scandinavian consensus recommendations for assessment and treatment of PSH during rehabilitation after severe acquired brain injury.

METHODS:

The CREDES recommendations(11) for Delphi processes were used as the basis for the consensus process.

Justification for use of a Delphi process: Uncertainties regarding best treatment for PSH and the lack of adequate research findings to assist physicians in clinical decisions were discussed at a meeting of the Brain Injury Pharmacotherapy Special Interest Group of the Nordic Network for Neurorehabilitation(12) at a meeting in Oslo in November 2022. A decision was taken to hold a Delphi process to establish the degree of consensus.

Planning and process: During the spring of 2023, the convenors (the authors) created a project plan and questions for the first round based on previous discussions of areas of difficulty and published, peer reviewed articles on PSH. These were discussed with an independent Rehabilitation physician and Neurosurgeon (see acknowledgements) from a different region to the convenors, following which adjustments were made to the project plan and questions. Before round one, Consultant physicians in Rehabilitation medicine at the authors' department, who were not participants in the panel, answered a pilot version and gave feedback on the question wording and content before final adjustments. The Delphi process was run between August and December 2023; Round one was distributed in August, round two in October and round three in December. An anonymised summary of responses was sent to the panel after completion of each round and before distribution of the next round.

Establishing the panel: Factors of importance regarding choice of panel participants were discussed by the convenors before sending invitations. Representation from each health care region in Denmark, Sweden and Norway was sought, with an aim of 1 to 3 panel participants from each region. We sought panel participants who were physicians who had completed specialist training, worked clinically with patients with severe acquired brain injury in the Nordic countries, and had been involved in clinical decisions about drugs treatment of PSH in the previous year; these formed criteria for participation. Potential panel participants were identified via lists of participants in the Nordic Network of Neurorehabilitation meeting in November 2022, by network contacts, and by contact with the authors of a recent Scandinavian publication on PSH(13). Invited participants received an email with information about the project including a reference to the key 2014 publication on consensus criteria, extent of panel involvement, and confirmed that they met the above criteria for panel participants prior to answering round one. Answers were in English or national languages.

Patient group: Patients with severe acquired brain injury defined as any sudden insult to a previously essentially healthy brain in a patient aged at least 2 years, of a severity that requires initial neurointensive care. Diagnoses include traumatic brain injury, anoxic brain injury, subarachnoid haemorrhage, spontaneous intracerebral haemorrhage, cerebral infarction.

Rehabilitation focus: The focus of this study was on patients undergoing rehabilitation after severe acquired brain injury, including early rehabilitation which may occur during the later stages of neurointensive care, intermediary care, care on an acute ward or inpatient care on a rehabilitation unit. Due to the focus on rehabilitation, and thereby the need for consideration of promotion of

For peer review only - http://bmjopen.bmj.com/site/about/guidelines.xhtml

recovery of awareness and cognition, this survey did not include patients who are anaesthetized and mechanically ventilated in the very early stages after brain injury; when other treatments may be necessary to minimize secondary brain injury and when such rehabilitation considerations are not yet relevant.

Definition of consensus: Round one was an exploratory round with the aim of finding an optimal focus for the rest of the process. The decision was therefore made (and communicated to panel participants) before round one that criteria for consensus would be decided after completion of round one. Panel participants received the following information on stopping criteria before they answered round two: "Statements that 75% of the panel agree with will be considered to represent consensus in the Scandinavian countries (Denmark, Sweden, Norway) at this time. The conveners will consider the development of responses and the likelihood of coming to consensus in specific aspects after round 3, and then consider whether further rounds (maximum 5) will give added value. If after 3 rounds we have not reached consensus for some items, and it is unlikely that consensus will be reached, areas where at least 50% of the panel agree will also be reported as areas of considerable agreement (but not consensus)."

Procedure for the rounds:

Round one included questions on panel participants' speciality and clinical experience, and on a wide range of aspects of PSH assessment and management. Questions were in various forms, where closed questions (possible answers, "yes, no, I don't know") were complemented with free text comment boxes. Round one also included questions about drugs used for treatment of PSH. A list was given based on 29 classes of drugs (with examples) reported as being used for PSH in a recent scoping review (4). For each phase of care that they participated in, panel participants were asked to indicate all drugs they used, also the three drugs most often used, and comment on reasons for their use or non-use. Phases of care were included due to expected differences in treatment priorities with care setting and were defined as follows: 1. Breathing spontaneously with no or only light sedation on the neurointensive care unit. The patient may have respiratory support. 2. Breathing spontaneously with no or only light sedation on an intermediate care unit / high dependency unit. The patient may have respiratory support. 3. Cared for on an acute care ward or rehabilitation ward at an acute hospital. The patient may have respiratory support. 4. Cared for on a rehabilitation ward without acute hospital facilities. 5. Cared for outside hospital (home, nursing home).

Round two: Based on collated responses to round one, a number of statements were created for round two, which the panel were asked to respond to with "agree, disagree" or, "I don't know". For each statement they could also comment and suggest changes. The five drugs that were included in at least 25% of the panels' lists of the three most commonly used drug treatments for PSH at any phase of care, were carried forward to round two. Panel participants were asked, for each phase of case which they participated in, to indicate how commonly they prescribed these drugs for PSH on a scale of 0 (never) to 50 (for about half of patients) to 100 (always).

Round three included reformulated statements for the minority of areas where consensus had not been reached in round two, with a new possibility to agree or disagree. Round three also included possible refinements for three statements where consensus had already been reached in round two, but where individual panel participants had suggested refinements or improvements in their responses to round two. Panel participants were in round three asked to confirm their agreement or otherwise with the original version of these statements from round two, state their agreement or otherwise to the adjusted versions of these statements, and finally to express a preference for one version.

Platform: REDCap(14) is a secure web application for building and managing online surveys and databases. REDCap was used to produce and send out the questionnaires for each round, to send pre-scheduled and extra reminders for submission of answers, and also to distribute summaries of

For peer review only - http://bmjopen.bmj.com/site/about/guidelines.xhtml

findings from each round before the following round. Personalised email reminders were also sent to panel participants who had not responded by the initial deadline, with extensions given of up to a week. Participants received no compensation for their participtation.

Ethics and Informed Consent: This study, in which participants were physicians contributing with their knowledge and opinions, did not fall with the scope of studies requiring ethical approval in Sweden (https://etikprovningsmyndigheten.se/en/) and as such ethical approval was not required. After receiving information about the study, panel participants clicked a response to the following question on the digital platform: "I give my consent to participate in the panel, for storage of my email address and de-identified responses according to GDPR for the duration of the Delphi-process, and for feedback of anonymized responses to the panel during the process. I also consent to publication of study findings". Possible responses were "yes" and "no". The digital platform was programmed so that the questionnaire was terminated if a "no" response was given, and access to the consensus questions was only available after a "yes" response.

RESULTS:

Panel participants: Of 23 physicians invited to participate, 20 met the criteria, formed the panel, and responded to rounds one and two. 19 of these also responded to round three. Four panel participants worked with patients in Denmark, seven panel participants in Norway and nine in Sweden. Medical specialities of panel participants were Rehabilitation Medicine or equivalent for 14, Intensive care medicine for two, Neurosurgery for three and neurology for one. Three of the Rehabilitation medicine specialists were also specialists in another area: two in neurology, and one in intensive care medicine. Panel participants had a mean of 10.3 years of clinical experience of acquired brain injury (standard deviation 6,7). Panel participants were from 2 of 4 Danish Health Care regions, 4 of 5 Norwegian health care regions and 5 of 6 Swedish Health care regions.

Panel participants' clinical involvement at the different phases of care: 6 panel participants were involved with decisions regarding PSH treatment for patients breathing spontaneously with no or light sedation on the neurointensive care unit, 10 on the intermediate care (or high dependency) unit, 12 during care on an acute or rehabilitation ward at an acute hospital, 9 during care on a rehabilitation ward without acute hospital facilities, and 3 for patients cared for outside hospital (at home or in an nursing home). Some panel participants were involved during several phases of care. The number of panel participants with current experience of patients care for outside hospital was considered too few for formation of consensus and this phase of care was not included in subsequent rounds.

Process: Consensus was reached for many areas in round 2. A third round was run to allow consideration of refinements of some statements and to consider alternatives to two statements where consensus had not been reached in round 2. The project was concluded at the end of round three.

Consensus statements: At least 75% of panel participants agreed to the following statements by the end of round three. Two statements concerned only the later phases of the chain of care. For these statements, only responses from panel participants who worked during the later phases of care were considered when calculating percentage agreement. Following each statement, the number of panel participants who agreed, disagreed or didn't know is given, and the round after which consensus was reached for the final wording is given.

1. The term paroxysmal sympathetic hyperactivity (PSH) is the preferred term for use in clinical practice. The diagnosis should be made according to the consensus definition of Baguley et al 2014. As described by Baguley et al, PSH may occur after severe acquired brain injury, including but not limited to traumatic brain injury. *Agree 19, Disagree 0, Don't know 1. Consensus after round 2.*

For peer review only - http://bmjopen.bmj.com/site/about/guidelines.xhtml

2. Differential diagnoses should be considered and as far as possible excluded and/or treated before making the diagnosis of PSH. *Agree 20, Disagree 0, Don't know 0. Consensus after round 2.*

3. The PSH assessment measure (PSH-AM) should be used to support the initial clinical diagnosis and management of PSH. In some clinical settings aspects of the PSH-AM may be difficult to implement and additional or alternative supporting tools may need to be developed. *Agree 17, Disagree 1, Don't know 2. Consensus after round 2.*

4. PSH will in most cases have developed and been diagnosed during neurointensive care or intermediary care. In those cases where the diagnosis has not been made, the PSH assessment measure is also recommended for use on acute wards or inpatient rehabilitation units. *Responses only sought from panel participants working on acute wards or inpatient rehabilitation units, n=14: Agree 14, Disagree 0, Don't know 0. Consensus after round 3.*

5. For patients who have a diagnosis of PSH made on the neurointensive or intermediary care ward and where drug treatment for PSH is ongoing when they are discharged, there is a need for follow up later in the chain of care. The PSH-assessment measure may be used during inpatient rehabilitation as part of follow up and to guide decisions on tapering of treatment. *Responses only sought from panel participants working on acute wards or inpatient rehabilitation units, n=14: Agree 14, Disagree 0, Don't know 0. Consensus after round 3.*

6.The effectiveness of prophylactic drug treatment for patients with a risk of PSH is unknown. Negative effects of drug treatment are difficult to evaluate in patients with severe acquired brain injury. At the current time prophylactic pharmacological treatment aimed at preventing PSH in patients who do not have a PSH diagnosis cannot therefore be recommended. *Agree 19, Disagree 0, Don't know 1. Consensus after round 2.*

7. General principles for treatment: Choice of PSH drug therapy should take into consideration phase of recovery, care setting, and current medical and rehabilitation priorities such as need to promote recovery of consciousness and cognition. *Agree 20, Disagree 0, Don't know 0. Consensus after round 2.*

8. When initiating treatment of PSH, treatment should aim to treat both sympathetic hyperactivity and to minimize the most prominent symptoms of PSH in the individual patient. *Agree 19, disagree 0, don't know 0. Consensus after round 3.*

9.PSH-triggers should be assessed, and measures taken to minimize these before and during any pharmacological treatment. PSH-triggers may include urinary retention, constipation, gastric paresis, spasticity, poor positioning (where spasticity may contribute), allodynic responses to poorly fitting orthotics, pressure sores and other causes of pain, environmental factors such as ambient temperature and noise. Units should develop routines for a structured approach to minimization of triggers. *Agree 20, Disagree 0, Don't know 0. Consensus after round 2.*

10. Supportive treatment (hydration, reduction of fever, correction of electrolytes, nutrition) should be optimized in parallel to pharmacological treatment for PSH. Delays in pharmacological treatment should be avoided in moderate to severe cases. Units should develop routines for a structured approach to optimization of supportive treatment. *Agree 17, Disagree 1, Don't know 1. Consensus after round 3.*

11. PSH as the treatment indication should be clearly documented in the medical record when prescribing drugs to treat PSH, when transferring patients between units, and at discharge. This is particularly important as many drugs used for PSH also have other indications. Treatment response to any current or previous PSH drug treatment should also be documented. Future medication review later in the chain of care should be planned. *Agree 19, Disagree 0. Don't know 1. Consensus after round 2.*

For peer review only - http://bmjopen.bmj.com/site/about/guidelines.xhtml

12. Sedating drugs should be minimized wherever possible when treating PSH, due to negative effects on recovery of consciousness and cognition, risk for other negative effects of sedation such as retained respiratory secretions, and impact on participation in rehabilitation. This is especially relevant in rehabilitation settings but should also be considered during neurointensive care and intermediary (high dependency) care. For very severe cases short periods of treatment with sedating drugs may be unavoidable. *After round 3: Agree 18, Disagree 1, Don't know 0. Consensus on a previous version in round 2, with final consensus on this alternative wording in round 3.*

13. The efficacy and relatively favourable side effect profiles of non-selective beta blockers and Gabapentin make these appropriate for first line treatment of PSH for most patients after neurointensive care. *After round 3: Agree 17, Disagree 0, Don't know 1, no answer 1. Consensus reached in round 2 and confirmed after rejection of an alternative wording in round 3.*

14. Opiates are often necessary for PSH early after injury due to good effects on many PSH components. However, they should be tapered when possible later during rehabilitation due to negative effects on alertness and cognition. *Agree 20, Disagree 0, Don't know 0. Consensus after round 2.*

15. Use of benzodiazepines primarily for treatment of PSH should be minimized (regarding dose and treatment duration) during phases in recovery when the focus is rehabilitation, due to negative effects on cognition and alertness. Their use may be necessary in the initial treatment of PSH on the neurointensive care unit and/or high dependency unit but they should be tapered when possible. *Agree 20, Disagree 0, Don't know 0. Consensus after round 2.*

16. Botulinum toxin may be needed for treatment of focal spasticity generally after severe acquired brain injury, and specifically for patients where focal or regional spasticity contributes to triggering of PSH-episodes. *Agree 19, Disagree 0, Don't know 1. Consensus after round 2.*

17. Enteral baclofen may be necessary for PSH with considerable disturbances of muscle tone, but negative effects on cognition and alertness may contribute to difficulties with participation in rehabilitation. These negative effects should be considered when prescribing and during follow up. *Agree 20, Disagree 0, Don't know 0. Consensus after round 2.*

18. An intrathecal baclofen pump should be considered for severe PSH where other measures have not been effective. *Agree 16, Disagree 1, Don't know 3. Consensus after round 2.*

19. PSH abates (reduces or disappears) with increased time after injury for most patients. Drug and other treatments for PSH should therefore be reviewed regularly and reduced or ceased if and when possible. *Agree 20, Disagree 0, Don't know 0. Consensus after round 2.*

20. The course of PSH in the post-acute phases (on the rehabilitation ward and beyond) and optimal tapering of medication when episodes abate are insufficiently documented and should be a focus of future research and development work. *Agree 15, Disagree 0, Don't know 2, No answer 3. Consensus after round 2.*

21. Ethical challenges in treating PSH in patients who often lack the capacity to consent to treatment are acknowledged. When the patient is unable to participate in treatment discussions next of kin and/or legal representatives should be informed of treatment decisions and involved in discussions of the often difficult balance between negative effects of PSH and potential negative effects of treatment (side effects, sedation). The presence of PSH is also associated with more severe brain injury with poorer outcomes, about which relatives should be informed. *Agree 19, Disagree 1, Don't know 0. Consensus after round 2.*

Choice of Drugs for PSH-treatment

For peer review only - http://bmjopen.bmj.com/site/about/guidelines.xhtml

In round one the panel indicated which drugs they use with a primary purpose of treating PSH. To support recall of any less usual choices, and to aid analysis, panel participants were presented with a list of 29 drug classes with examples from a 2021 Scoping Review on PSH(4) and asked to indicate all drugs they used: there was an unlimited number of choices, and no restrictions were made at this stage regarding phase of recovery. Drugs used were (drug, number of panel participants): Opiates (15), non-selective β-blockers e.g. Propranolol (14), Gabapentionoid e.g. Gabapentin (14), GABA-B agonist e.g. Baclofen (11), α2 agonist e.g. Clonidine (10), Paracetamol (10), selective β-blockers (4), antiepileptics e.g. Carbamazapine, Valproate (3), antihistamine e.g. Hydroxyzine (3), Benzodiazepines e.g. Diazepam (3), Other anesthetics e.g. Propofol (3), Adamantane e.g. Amantadine (2) Sedative hypnotics e.g. Zopiclone (2), Barbiturates e.g. Phenobarbital (1), Dopamine agonist e.g. Bromocriptine (1), Hyrdrazinophalazine e.g. Didhydralazine (1), Ibuprofen (1), Other - Muscle relaxant (1), Others (0).

In round two, panel participants indicated how often they used the drugs that had been most commonly cited in round one, for each of the phases of care in which they were involved (see table), by moving a horizontal slider for each drug. The five drug classes for which responses were sought, and given in the table, were those which in round one were included in at least 25% of individual panel participants' lists of their three most used drugs, at any stage of care.

------ *insert table near here* -------

For peer review only

Algorithm: The consensus statements and panel particpants' drug choices are summarised in the algorithm in the figure, which is intended to support clinicians in their assessment and management of patients with PSH. The algorithm was presented to the panel in round three with resulting comments incorporated into the final version --------- *insert figure near here* ---------.

DISCUSSION:

After an initial exploratory round in this Delphi process on the management of PSH during rehabilitation after severe acquired brain injury, consensus was reached for 18 statements in round two, on terminology, assessment, non-pharmacological management, and treatment principles. Consensus for three further statements and refined wording for two statements were achieved in round three.

Responses on drug choices were less clear cut: panel participants' drug choices at the various phases of care show clear trends, but also a considerable variability in practice. This was not unexpected given the weak state of the evidence (4). Alpha-2 agonists and opiates are used most in the early stages of recovery, on the neurointensive care and intermediary care units, and infrequently later during inpatient care and rehabilitation, although some panel participants continue to use these drugs even at these later stages. Non-selective beta-blockers are most commonly used at all phases of care after neurointensive care, and Gabapentin is also commonly used in these phases.

These drug choices are consistent with targets suggested by current theoretical models (5,7) that focus on failure of central inhibition of sympathetic and motor responses, mediated by high levels of catecholamines during episodes. Alpha-2 agonists such as Clonidine reduce sympathetic outflow but also cause sedation. Opiates are effective against pain responses, but unwanted effects including sedation, respiratory depression, and cognitive effects become relevant as rehabilitation progresses. The non-selective beta blocker Propranolol is both cardioprotective and neuroprotective, reduces the effects of high catecholamine levels and reduces metabolic rate (15). A recent single center randomised controlled study of propranolol and clonidine for severe traumatic brain injury however found no effect on outcome (16); this study was however not restricted to patients with PSH so the relevance of the finding is unclear. Gabapentin acts on voltage dependent calcium channels in the dorsal horns of the spinal cord to inhibit neuropathic pain (17), and as such may decrease the uncontrolled nociceptive or allodynic drive to PSH, and can be safely used without continuous monitoring.

Trials to date are limited and do not give sufficient evidence on which to base specific drug choices during an individual patient's recovery and rehabilitation; the consensus statements developed through this Delphi process likely represent clinical syntheses by the panel of a broad range of parameters related to the holistic rehabilitation process in coming to decisions about positive and negative aspects of particular drugs, in the face of insufficient evidence.

Several drugs on the list provided to panel members from the scoping review (4) were used by no or only one panel member; generally these were, as expected, drugs with the weakest of evidence of and/or putative mechanisms for effect, for example Hydralazine, Angiotension 2 antagonists, Phenothiazines. However, the dopamine agonist Bromocriptine, considered to target hyperpyrexia and sweating, with a reasonable theoretical basis and more extensive (although still weak) evidence base was also only used by a single panel member. Reasons for this non-use of Bromocriptine were not further investigated, but could include side effect profile, safety considerations in care settings without continuous monitoring, and/or lack of familiarity.

Panel feedback also highlighted certain management difficulties due to non-patient factors. For example, although consensus was reached that an intrathecal baclofen pump should be considered for the most severe, refractory cases, this may be unavailable in some centres. Whilst intrathecal

For peer review only - http://bmjopen.bmj.com/site/about/guidelines.xhtml

baclofen is well established for the treatment of severe spasticity without PSH, and case reports and series exist dating back 20 years (see (18) for summary), the quality of evidence remains poor for this potentially effective but invasive intervention.

A number of the consensus statements are of a confirmatory nature, reiterating key aspects of the literature on PSH and putting these into a clinical context for routine practice. For example, although it will soon be 10 years since the publication of consensus on terminology and diagnosis, initial discussions and feedback to round one showed that other terms remain in use in today's clinical practice in the Scandinavian countries. Some statements also make explicit aspects that might be considered obvious components of good care, but where panel particpants' clinical experiences are of common difficulties (for example non-tapering of drugs as PSH abates). It is hoped that this broad Scandinavian consensus on recommended terminology, assessment and treatment principles will support good clinical practice for patients with PSH. Other consensus statements begin to address common difficulties encountered in later clinical management of patients who may need to be transferred between units several times before leaving inpatient care. The panel's experience is that indications for each prescribed drug are not always specified at the time of transfer between units, which creates difficulties in follow up and ongoing optimisation of drug treatment.

The consensus process was guided by the initial exploratory round and focused on areas prioritised during neurorehabilitation. Other areas of clinical uncertainty exist where further guidance would aid clinicians. One example is difficulties in applying current diagnostic criteria when suspicion of PSH co-exists with a (suspected or diagnosed) complication that could in part explain PSH-like symptoms. PSH is currently a diagnosis of exclusion (1), and the PSH-AM does not allow for such a clinical scenario.

Strengths of the consensus process include the following of published recommendations for Delphi processes, an explorative first round, clear differentiation in questions to panel participants of the various phases of care (from neurointensive care to a rehabilitation ward outside an acute hospital), inclusion of panel participants from several medical specialities, inclusion of non-pharmacological aspects of assessment and management, and broad geographical representation from Denmark, Norway and Sweden. Limitations include an insufficient number of panel participants with experience of care after discharge from inpatient rehabilitation for consideration of this phase, and lack of data on the extent of panel participant's clinical experience of PSH; although it was a requirement that panel participants had been involved in clinical decisions for patients with PSH during the past year, there was no specific requirement regarding the number of patients treated by panel participants. We acknowledge difficulties in defining the start point for these recommendations; the description used, "breathing spontaneously with no or light sedation on the neurointensive care unit", was developed to portray concisely to the panel a patient who has survived initial neurosurgical interventions and is entering a phase where rehabilitation considerations such as recovery of awareness and cognition begin to become relevant and where sedating drugs may potentially adversely affect such processes. We did not however further define "light sedation".

The limitations of a Delphi process representing expert opinion, should also be noted: studies with randomised, controlled methodologies are needed to provide evidence-based recommendations. These should be based as far as possible on knowledge of underlying PSH-mechanisms, and ideally include patients during all stages of care, with long term follow up. However, given the clinical complexity of patients with PSH, logistical and ethical barriers to such studies are considerable. We consider that this Delphi process adds to previous studies by giving more explicit consideration to changing priorities through the rehabilitation chain of care and clarifies the necessity of an interdisciplinary approach to assessment and management involving the rehabilitation team, at all phases including very early rehabilitation. Identifiable triggers were noted in a high proportion (72%)

For peer review only - http://bmjopen.bmj.com/site/about/guidelines.xhtml

of patients in one study (7). The panel's recommendation of a systematic interdisciplinary approach to identifying and minimising these triggers seems justified.

An algorithm has been included (see figure) as a summary of the consensus statements, with the intention of assisting physicians in treatment of patients with PSH in all phases of inpatient care after early sedation on the neurointensive care unit has been reduced.

Whilst robust research studies of the effects of drugs on PSH are desirable, the clinical complexity of most patients affected by PSH and its relative rarity are expected to make definitive studies difficult to perform. Studies evaluating real-world data from treatment of patients carried out during routine clinical practice are also needed to improve treatment. For this to be possible, better structures are needed for assessment, diagnosis, treatment and follow up. "Structures" include routines guided by these consensus criteria, and also consideration of medical journal systems and coding; there is no ICD-10 diagnosis for paroxysmal sympathetic hyperactivity which is a hinder to data assimilation from clinical practice.

One problem with current treatment strategies is that they are of necessity reactive. Ideally early, sub-clinical signs of PSH would be identified. Treatment of such preclinical stages would have theoretical benefits. Mathematical modelling of continuous patient data (6) is an area of emerging interest together with machine learning and has the potential to aid early identification and treatment of PSH. Extending such methods to phases of care where continuous vital parameter monitoring is no longer available is a further challenge.

There are also examples of limitations in electronic medical journal systems preventing documentation of paroxysmal sympathetic hyperactivity as a drug indication as the term has more characters than allowed in some prescribing systems. Such organisational and system factors should receive more attention in future studies and planning for care of patients with severe acquired brain injury. Continued educational efforts about PSH are also needed to inform the less specialised health care staff that the patient is likely to encounter with increased time after brain injury.

CONCLUSION:

A considerable degree of consensus has been reached in Scandinavian countries for the clinical assessment and management of patients with PSH after severe acquired brain injury. Uncertainties remain about specific drug choices, which need to be modified in pace with changing rehabilitation priorities.

REFERENCES:

1. Baguley IJ PI, Fernandez-Ortega JF, Rabinstein AA, Dolce G, Hendricks HT. Paroxysmal sympathetic hyperactivity after acquired brain injury: consesnsus on conceptual definition, nomenclature, and diagnostic criteria. Journal of Neurotrauma. 2014;1;31(17):1515-20.
2. Geert Meyfroidt IJB, David K Menon. Paroxysmal sympathetic hyperactivity: the storm after acute brain injury. Lancet Neurology. 2017;16(9):721-9.
3. Rui-Zhe Zheng Z-QL, Run-Ze Yang, Guo-Hui Huang, Guang-Ming Zhang. Identification and Management of Paroxysmal Sympathetic Hyperactivity After Traumatic Brain Injury. Frontiers of Neurology. 2020;11:81.
4. Justin Siew Yoong Tu JR, Adam M Deane, Mark P Plummer. Pharmacological Management of Paroxysmal Sympathetic Hyperactivity: A Scoping Review. Journal of Neurotrauma. 2021;38(16):2221-37.

For peer review only

For peer review only - http://bmjopen.bmj.com/site/about/guidelines.xhtml

5. Ian J. Baguley, The excitatory:inhibitory ratio model (EIR model): An integrative explanation of acute autonomic overactivity syndromes, Medical Hypotheses, 2008-01-01, Volume 70, Issue 1, Pages 26-35

6. Podell J, Pergakis M, Yang S, *et al,* Leveraging Continuous Vital Sign Measurements for Real-Time Assessment of Autonomic Nervous System Dysfunction After Brain Injury: A Narrative Review of Current and Future Applications. Neurocrit Care. 2022 Aug;37(Suppl 2):206-219. doi: 10.1007/s12028-022-01491-6. Epub 2022 Apr 12.

7. Fernandez-Ortega JF, Baguley IJ, Gates TA, *et al,* Catecholamines and Paroxysmal Sympathetic Hyperactivity after Traumatic Brain Injury, Neurotrauma. 2017 Jan 1;34(1):109-114. doi: 10.1089/neu.2015.4364. Epub 2016 Jul 8.

8. Zhu K, Zhu Y, Hou X, *et al,* NETs Lead to Sympathetic Hyperactivity After Traumatic Brain Injury Through the LL37-Hippo/MST1 Pathway, Front Neurosci. 2021 Apr 29:15:621477. doi: 10.3389/fnins.2021.621477. eCollection 2021.

9. Sun, Miao, Wang *et al* Decreased levels of hydrogen sulfide in the hypothalamic paraventricular nucleus contribute to sympathetic hyperactivity induced by cerebral infarction. Hypertens Res. 2024 Mar 15. doi: 10.1038/s41440-024-01643-5.

10. Lucia F Lucca ADT, Francesca Cava, Annamaria Romoli, Rita Formisano, Federico Scarponi, Anna Estraneo, Diana Frattini, Paolo Tonin, Chiara Bertolino, Pamela Salucci, Bahia Hakiki, Mariagrazia D'Ippolito, Mauro Zampolini, Orsola Masotta, Silvia Premoselli, Matteo Interlenghi, Christian Salvatore, Annalisa Polidori, Antonio Cerasa. Predicting Outcome of Acquired Brain Injury by the Evolution of Paroxysmal Sympathetic Hyperactivity Signs. Journal of Neurotrauma. 2021;38:1988-94.

11. Saskia Jünger SAP, Jenny Brine, Lukas Radbruch and Sarah G Brearley. Guidance on Conducting and REporting DElphi Studies (CREDES) in palliative care: Recommendations based on a methodological systematic review. Palliative Medicine. 2017.

12. Nordic Network for Neurorehabilitation . [19th January 2024]. Available from: https://www.nordisk-netvaerk-neurorehab.com/formaal/.

13. Alba Corell JL. ABC om Paroxysmal sympatisk hyperaktivitet (Article in Swedish). Läkartidningen. 2021;118:21070.

14. RedCap 2024 [updated 19th January 2024. Available from: https://www.project-redcap.org/.

15. Stéphane Nguembu, Marco Meloni, Geneviève Endalle, Hugues Dokponou, Olaoluwa Ezekiel Dada, Wah Praise Senyuy, Ulrick Sidney Kanmounye, Paroxysmal Sympathetic Hyperactivity in Moderate-to-Severe Traumatic Brain Injury and the Role of Beta-Blockers: A Scoping Review, Emerg Med Int. 2021 Sep 11:2021:5589239. doi: 10.1155/2021/5589239. eCollection 2021.

16. Nordness MF, Maiga AW,Wilson LD, *et al* Effect of propranolol and clonidine after severe traumatic brain injury: a pilot randomized clinical trial Crit Care. 2023 Jun 9;27(1):228. doi: 10.1186/s13054-023-04479-6.

17. Ian J Baguley, Roxana E Heriseanu, Joseph A Gurka, Annette Nordenbo, and Ian D Cameron, Gabapentin in the management of dysautonomia following severe traumatic brain injury: a case series, J Neurol Neurosurg Psychiatry. 2007 May; 78(5): 539–541.doi:10.1136/jnnp.2006.096388

18. Pucks-Faes E, Hitzenberger G, Matzak H, *et al*. Intrathecal baclofen in paroxysmal sympathetic hyperactivity: Impact on oral treatment, Brain Behav. 2018 Nov;8(11):e01124. doi: 10.1002/brb3.1124. Epub 2018 Sep 28.

For peer review only - http://bmjopen.bmj.com/site/about/guidelines.xhtml

Author contributions: All authors contributed to study design, conduction of the study, and analysis of data. Drs Zampakas and Deboussard formed and distributed the surveys in REDCap. Dr Godbolt drafted the article and created a sketch for the figure. Drs Zampakas and Deboussard critically appraised and approved the article text.

Acknowledgments - We thank the following:

Doctors Per Ertzgaard, Rehabilitation Physician and Fredrik Ginstman, Neurosurgeon, both of the University Hospital Linköping, Sweden, for valuable comments on the project plan and a pilot version of the questions for round one.

The panel participants for their valuable contributions to the Delphi Consensus process: Daniel Dahlgren and Alison Godbolt, Department of Rehabilitation Medicine, Danderyd Hospital, Sweden. Sofie Jacobson, Department of Rehabilitation Medicine, Umeå University Hospital, Sweden. My Dung Nguyen Torkildsen, Head of department, Physical medicine and rehabilitation, Stavanger University Hospital, Norway. Tanja Bertheussen, Dept of Physical medicine and Rehabilitation, Kongsgård, Sørlandet Hospital, Norway. Alba Corell, Department of Clinical Neuroscience, Institute of Neuroscience and Physiology, Sahlgrenska Academy, University of Gothenburg, Gothenburg, Sweden, Department of Neurosurgery, Sahlgrenska University Hospital, Gothenburg, Sweden. Kristin Alvsaaker, Postoperative and Intensive Care Department, Oslo University Hospital (OUH), Norway and Department of Physical Medicine and Rehabilitation, OUH, Norway. Tiina Ader, Department of Physical Medicine and Rehabilitation, Haukeland University Hospital, Norway. Merete Petersen, Department of Neuroanesthesiology, Rigshospitalet, University Hospital, Denmark. Cathrine Elisabeth Einarsen, Clinic of Rehabilitation, St. Olavs Hospital, Trondheim University Hospital, Trondheim, Norway. Tove Grønbæk Jensen, Hammel neurocentre, Denmark. Marina Parziali, Neurorehabilitation, NU Health Care, Sweden. Ursula Heldmann, Rehabilitation medicine, Skane University Hospital, Sweden. Astrid Wille-Jørgensen, Dept. of Brain and Spinal Cord Injury, Bodil Eskesen Center, Rigshospitalet, Demark. Maritza Beekman, Department of Rehabilitation Medicine, University Hospital Linköping, Sweden. Marco Brizzi, Senior Consultant Neurologist, Neuro Intermediate Care Unit, Malmoe, Dept of Neurology and Rehabilitation medicine, Skane University Hospital, Sweden. Caroline Ustvedt, Department of Traumatic Brain Injury, Sunnaas Rehabilitation Hospital, Norway. Shirin Kordasti Frisvold, Chief consultant, Department of Intensive Care and Department of Anesthesiology and Intensive Care, University Hospital of North Norway, Tromso. Rasmus Langelund Jørgensen, Hammel Neurorehabilitation and Research Centre, Denmark. Laura Serrano Barrenechea, Dept of Rehabilitation Medicine South Älvsborgs Hospitla and Ryhov Hospital.

Stefan Arousell, Dept of Rehabilitation Medicine, Danderyd hospital, Stockholm for graphic design support in creation of the figure.

Data availability statement: Deidentified data (panel responses) are available upon reasonable request to the corresponding author on presentation of a valid research question from an established researcher.

Funding statement: This research received no specific grant from any funding agency in the public, commercial or not-for-profit sectors.

Patient and public involvement: Patients and the public were not involved.

Competing Interest Statement: Dr Godbolt is the specialty expert for Rehabilitation Medicine for the Stockholm Region. Dr Zampakas has received compensation from Ipsen for support with a workshop at the Nordic Toxins conference, October 2023. Dr Deboussard is the Chair for the National Program

Group, and for the Regional Program Group for Stockholm-Gotland, for Rehabilitation Habilitation and Insurance Medicine, within the National system for knowledge-driven management within Swedish healthcare.

Ethical Approval Statement: Ethical approval was not required as no patients were involved. Panel participants gave consent for participation in the panel, storage of email addresses and de-identified responses, feedback of anonymised responses to the panel during the process, and publication of study findings.

For peer review only

Table: Panel participants' frequency of use of common drugs for PSH

	Neurointensive care	Intermediary care	Acute Hospital – acute or rehabilitation ward	Inpatient rehabilitation outside acute hospital
respondents	*n=6*	*n=9*	*n=9*	*n=8*
**Drug:**				
Alpha-2 agonists e.g. Clonidine	80,5 [30 – 100]	50 [16-100]	23,5 [0-96]	10 [0-90]
Baclofen (enteral)	32,5 [0-89]	26,5 [0-75]	22 [0-70]	28 [12-50]
Beta blocker, non-selective, e.g. Propranolol	65 [20-94]	83 [16-100]	81 [50-100]	75,5 [50-100]
Gabapentin	38 [0-63]	50 [0–93]	60,5 [21-90]	50 [27-94]
Opiates	75 [64-85]	68 [31–99]	26 [0-70]	28 [0-96]

Legend for table: The table gives the median and [range] for how often panel members use the stated drugs to treat PSH, on a continuous scale where 0 = never, 50= half of patients, 100 = always. Note: Data characteristic suggested technical difficulties for 2 panel participants: in one case "50" was registered for all drugs in all care settings, which seems unlikely to be the intended response. In another case 0 was registered for all drugs for all care settings after intermediary care; the instruction was to not answer for care settings that respondents do not work in rather than to answer 0. These responses have been excluded from the summary above.

Figure – see separate file

Legend for figure: *PSH – paroxysmal sympathetic hyperactivity, CRS-R – coma recovery scale-revised*

For peer review only

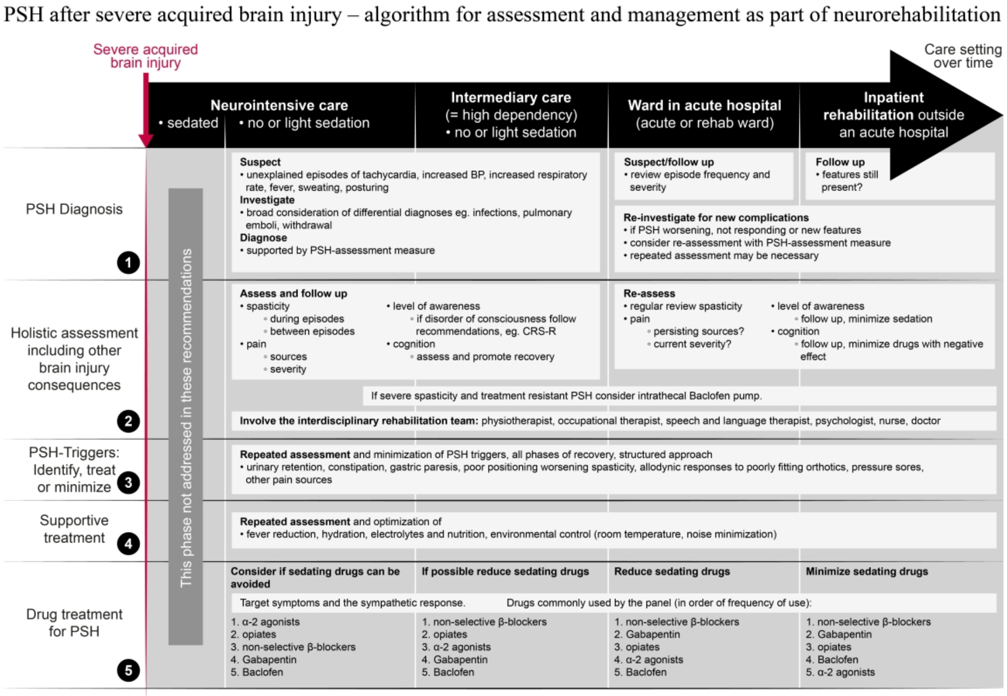

PSH – paroxysmal sympathetic hyperactivity, CRS-R – coma recovery scale-revised

268x188mm (500 x 500 DPI)

Checklist for submission to BMJ Open

Box 3. Recommendations for the Conducting and REporting of DElphi Studies (CREDES).

Rationale for the choice of the Delphi technique

1. *Justification.* The choice of the Delphi technique as a method of systematically collating expert consultation and building consensus needs to be well justified. When selecting the method to answer a particular research question, it is important to keep in mind its constructivist nature Included

Planning and design

2. *Planning and process.* The Delphi technique is a flexible method and can be adjusted to the respective research aims and purposes. Any modifications should be justified by a rationale and be applied systematically and rigorously Noted
3. *Definition of consensus.* Unless not reasonable due to the explorative nature of the study, an a priori criterion for consensus should be defined. This includes a clear and transparent guide for action on (a) how to proceed with certain items or topics in the next survey round, (b) the required threshold to terminate the Delphi process and (c) procedures to be followed when consensus is (not) reached after one or more iterations Done

Study conduct

4. *Informational input.* All material provided to the expert panel at the outset of the project and throughout the Delphi process should be carefully reviewed and piloted in advance in order to examine the effect on experts' judgements and to prevent bias Done
5. *Prevention of bias.* Researchers need to take measures to avoid directly or indirectly influencing the experts' judgements. If one or more members of the research team have a conflict of interest, entrusting an independent researcher with the main coordination of the Delphi study is advisable No conflict of interest of relevance

*Consolidated criteria for reporting qualitative research
**Consolidated Standards of Reporting Trials

Box 3. (Continued)

6. *Interpretation and processing of results.* Consensus does not necessarily imply the 'correct' answer or judgement; (non)consensus and stable disagreement provide informative insights and highlight differences in perspectives concerning the topic in question Noted
7. *External validation.* It is recommended to have the final draft of the resulting guidance on best practice in palliative care reviewed and approved by an external board or authority before publication and dissemination No such body available.

Reporting

8. *Purpose and rationale.* The purpose of the study should be clearly defined and demonstrate the appropriateness of the use of the Delphi technique as a method to achieve the research aim. A rationale for the choice of the Delphi technique as the most suitable method needs to be provided Done
9. *Expert panel.* Criteria for the selection of experts and transparent information on recruitment of the expert panel, socio-demographic details including information on expertise regarding the topic in question, (non)response and response rates over the ongoing iterations should be reported Done
10. *Description of the methods.* The methods employed need to be comprehensible; this includes information on preparatory steps (How was available evidence on the topic in question synthesised?), piloting of material and survey instruments, design of the survey instrument(s), the number and design of survey rounds, methods of data analysis, processing and synthesis of experts' responses to inform the subsequent survey round and methodological decisions taken by the research team throughout the process Done
11. *Procedure.* Flow chart to illustrate the stages of the Delphi process, including a preparatory phase, the actual 'Delphi rounds', interim steps of data processing and analysis, and concluding steps Covered by article text.
12. *Definition and attainment of consensus.* It needs to be comprehensible to the reader how consensus was achieved throughout the process, including strategies to deal with non-consensus Done.
13. *Results.* Reporting of results for each round separately is highly advisable in order to make the evolving of consensus over the rounds transparent. This includes figures showing the average group response, changes between rounds, as well as any modifications of the survey instrument such as deletion, addition or modification of survey items based on previous rounds Done
14. *Discussion of limitations.* Reporting should include a critical reflection of potential limitations and their impact of the resulting guidance Done
15. *Adequacy of conclusions.* The conclusions should adequately reflect the outcomes of the Delphi study with a view to the scope and applicability of the resulting practice guidance Done
16. *Publication and dissemination.* The resulting guidance on good practice in palliative care should be clearly identifiable from the publication, including recommendations for transfer into practice and implementation. If the publication does not allow for a detailed presentation of either the resulting practice guidance or the methodological features of the applied Delphi technique, or both, reference to a more detailed presentation elsewhere should be made (e.g. availability of the full guideline from the authors or online; publication of a separate paper reporting on methodological details and particularities of the process (e.g. persistent disagreement and controversy on certain issues)). A dissemination plan should include endorsement of the guidance by professional associations and health care authorities to facilitate implementation Dissemination planned.

ref: Guidance on Conducting and REporting DElphi Studies (CREDES) in palliative care. Jünger et al, Palliative Medicine 2017

1/2

ACCORD checklist for bmjopen-2024-084778 revision
Godbolt, Zampakas, Deboussard

Table 4. The final ACCORD checklist for the reporting of consensus methods.

Item number	Manuscript section	Item wording	Help text	line (marked copy)
T1	Title	Identify the article as reporting a consensus exercise and state the consensus methods used in the title.	For example, Delphi or nominal group technique.	2
I1	Introduction	Explain why a consensus exercise was chosen over other approaches.	n/a	108-112
I2	Introduction	State the aim of the consensus exercise, including its intended audience and geographical scope (national, regional, global).	n/a	103-104
I3	Introduction	If the consensus exercise is an update of an existing document, state why an update is needed, and provide the citation for the original document.	n/a	N/A
M1	Methods > Registration	If the study or study protocol was prospectively registered, state the registration platform and provide a link. If the exercise was not registered, this should be stated.	Recommended to include the date of registration.	Not registered
M2	Methods > Selection of SC and/or panelists	Describe the role(s) and areas of expertise or experience of those directing the consensus exercise.	For example, whether the project was led by a chair, co-chairs, or a steering committee, and, if so, how they were chosen. List their names, if appropriate, and whether there were any subgroups for individual steps in the process.	113
M3	Methods > Selection of SC and/or panelists	Explain the criteria for panelist inclusion and the rationale for panelist numbers. State who was responsible for panelist selection.	n/a	124 – 132
M4	Methods > Selection of SC and/or panelists	Describe the recruitment process (how panelists were invited to participate).	Include communication/advertisement method(s) and locations, numbers of invitations sent, and whether there was centralized oversight of invitations or if panelists were asked/allowed to suggest other members of the panel.	132 – 134
M5	Methods > Selection of SC and/or panelists	Describe the role of any members of the public, patients, or carers in the different steps of the study.	n/a	571
M6	Methods > Preparatory research	Describe how information was obtained prior to generating items or other materials used during the consensus exercise.	This might include a literature review, interviews, surveys, or another process.	165
M7	Methods > Preparatory research	Describe any systematic literature search in detail, including the search strategy and dates of search or the citation if published already.	Provide the details suggested by the PRISMA reporting guideline and the related PRISMA-Search extension.	N/A
M8	Methods > Preparatory research	Describe how any existing scientific evidence was summarized and if this evidence was provided to the panelists.	n/a	133
M9	Methods > Assessing consensus	Describe the methods used and steps taken to gather panelist input and reach consensus (for example, Delphi, RAND/UCLA, nominal group technique).	If modifications were made to the method in its original form, provide a detailed explanation of how the method was adjusted and why this was necessary for the purpose of your consensus-based study.	159
M10	Methods > Assessing consensus	Describe how each question or statement was presented and the response options. State whether panelists were able to or required to explain their responses, and whether they could propose new items.	Where possible, present the questionnaire or list of statements as supplementary material.	161 – 167, 175, 181-88
M11	Methods > Assessing consensus	State the objective of each consensus step.	A step could be a consensus meeting, a discussion or interview session, or a Delphi round.	159 – 188
M12	Methods > Assessing consensus	State the definition of consensus (for example, number, percentage, or categorical rating, such as "agree" or "strongly agree") and explain the rationale for that definition.	n/a	148-158
M13	Methods > Assessing consensus	State whether items that met the prespecified definition of consensus were included in any subsequent voting rounds.	n/a	181 - 188
M14	Methods > Assessing consensus	For each step, describe how responses were collected, and whether responses were collected in a group setting or individually.	n/a	189 - 194
M15	Methods > Assessing consensus	Describe how responses were processed and/or synthesized.	Include qualitative analyses of free-text responses (for example, thematic, content, or cluster analysis) and/or quantitative analytical methods, if used.	159 →

(Continued)

N/A = not applicable

ACCORD Checklist continued 2/2
bmjopen-2024-084778 revision
Godbolt, Zampakas, Deboussard

Table 4. (Continued)

Item number	Manuscript section	Item wording	Help text	line (marked copy)
M16	Methods > Assessing consensus	Describe any piloting of the study materials and/or survey instruments.	Include how many individuals piloted the study materials, the rationale for the selection of those individuals, any changes made as a result, and whether their responses were used in the calculation of the final consensus. If no pilot was conducted, this should be stated.	117-120
M17	Methods > Assessing consensus	If applicable, describe how feedback was provided to panelists at the end of each consensus step or meeting.	State whether feedback was quantitative (for example, approval rates per topic/item) and/or qualitative (for example, comments, or lists of approved items), and whether it was anonymized.	191-2
M18	Methods > Assessing consensus	State whether anonymity was planned in the study design. Explain where and to whom it was applied and what methods were used to guarantee anonymity.	n/a	121
M19	Methods > Assessing consensus	State if the steering committee was involved in the decisions made by the consensus panel.	For example, whether the steering committee or those managing consensus also had voting rights.	540
M20	Methods > Participation	Describe any incentives used to encourage responses or participation in the consensus process.	For example, whether invitations to participate reiterated, or were participants reimbursed for their time.	192-4
M21	Methods > Participation	Describe any adaptations to make the surveys/meetings more accessible.	For example, the languages in which the surveys/meetings were conducted and whether translations or plain language summaries were available.	135
R1	Results	State when the consensus exercise was conducted. List the date of initiation and the time taken to complete each consensus step, analysis, and any extensions or delays in the analysis.	n/a	111-121
R2	Results	Explain any deviations from the study protocol, and why these were necessary.	For example, addition of panel members during the exercise, number of consensus steps, stopping criteria; report the step(s) in which this occurred.	201-202
R3	Results	For each step, report quantitative (number of panelists, response rate) and qualitative (relevant sociodemographics) data to describe the participating panelists.	n/a	201-202<br>204-209
R4	Results	Report the final outcome of the consensus process as qualitative (for example, aggregated themes from comments) and/or quantitative (for example, summary statistics, score means, medians, and/or ranges) data.	n/a	223-321
R5	Results	List any items or topics that were modified or removed during the consensus process. Include why and when in the process they were modified or removed.	n/a	219-222
D1	Discussion	Discuss the methodological strengths and limitations of the consensus exercise.	Include factors that may have affected the decisions (for example, response rates, representativeness of the panel, potential for feedback during consensus to bias responses, potential impact of any nonanonymized interactions).	411-427
D2	Discussion	Discuss whether the recommendations are consistent with any preexisting literature and, if not, propose reasons why this process may have arrived at alternative conclusions.	n/a	360-385
O1	Other information	List any endorsing organizations involved and their role.	n/a	110-112
O2	Other information	State any potential conflicts of interests, including among those directing the consensus study and panelists. Describe how conflicts of interest were managed.	n/a	572-577
O3	Other information	State any funding received and the role of the funder.	Specify, for example, any funder involvement in the study concept/design, participation in the steering committee, conducting the consensus process, funding of any medical writing support. This could be disclosed in the methods or in the relevant transparency section of the manuscript. Where a funder did not play a role in the process or influence the decisions reached, this should be specified.	569

UCLA, University of California, Los Angeles

ACCORD, ACcurate COnsensus Reporting Document; n/a, not applicable; PRISMA, Preferred Reporting Items for Systematic Reviews and Meta-Analyses; SC, Steering Committee.

https://doi.org/10.1371/journal.pmed.1004326.t004

For peer review only - http://bmjopen.bmj.com/site/about/guidelines.xhtml
